# The impact of the intensity of media use on potential tourists’ risk perception and travel protective behavioral intentions in COVID-19

**DOI:** 10.3389/fpsyg.2023.1201481

**Published:** 2023-08-29

**Authors:** Ruihong Sun, Xinliang Ye, Jianping Tang, Jiexi Yang, Noel Scott

**Affiliations:** ^1^Shanghai University of Engineering Sciences, Shanghai, China; ^2^Department of Psychology, Faculty of Arts, University of British Columbia, Vancouver, BC, Canada; ^3^Sustainability Research Centre, University of the Sunshine Coast, Sunshine Coast, QLD, Australia; ^4^Faculty of Business and Law, Edith Cowan University, Edith Cowan, WA, Australia

**Keywords:** new media, traditional media, media use intensity, epidemic risk perception, travel protective behavioral intention, COVID-19 impact

## Abstract

**Introduction:**

In light of the COVID-19 pandemic, there is an increased need for potential travelers to gather information about their trips to mitigate perceived risks. This study aims to understand the relationship between the intensity of media use (both new and traditional), epidemic risk perception, and tourism protection behavior intention among potential tourists.

**Methods:**

A total of 491 valid questionnaires were collected in Shanghai, China. Factor analysis, path analysis, and effect analysis were conducted using SPSS and AMOS to examine the impact of different media types on epidemic risk perception and tourism protection behavior.

**Results:**

The findings indicate a positive association between new media use intensity and epidemic risk perception, as well as an intention to adopt safety-conscious tourism behaviors. In contrast, traditional media usage is inversely associated with risk perception but has no significant influence on protective behavior. The results also highlight the role of demographic factors, such as age, education level, occupation, and income, in modulating the relationship between media usage and risk perception.

**Discussion:**

The contrasting effects of new and traditional media suggest the need for a tailored approach in epidemic communication strategies. Public health officials should leverage new media to enhance risk perception and safety-oriented behaviors, while recognizing the role of traditional media in managing lower risk perceptions and assuaging panic. The study emphasizes the importance of personalized messaging based on demographic disparities in media usage and perception. The mediating role of risk perception in shaping protective behaviors offers insights for promoting adherence to safety protocols.

**Conclusion:**

This study contributes to a comprehensive understanding of media influences during health crises, emphasizing the responsibility of media platforms in transmitting accurate information. The findings call for a nuanced approach to epidemic communication, considering the strengths and weaknesses of different media types. Segmented and personalized messaging strategies can cater to demographic variations in media usage and perception. Enhancing risk perception through tailored messaging can promote protective behaviors and effectively manage public sentiment during health crises.

## 1. Introduction

The COVID-19 pandemic significantly altered travel behaviors and attitudes worldwide (Johnson et al., 2023). Since the global outbreak of the virus in February 2020, tourism has come to be viewed as a high-risk activity (Seyfi et al., 2020). The mobility and interaction of visitors have notably propagated the disease (Gibbs et al., 2020; Wen et al., 2021). As a result, most countries implemented measures such as travel restrictions, border closures, entry and exit bans, visa restrictions, and flight suspensions to curtail disease spread (Liebig et al., 2021).

Moreover, the outbreak of COVID-19 and subsequent lockdowns increased public reliance on various media outlets (Courbet et al., 2022). The demand for information about COVID-19 escalated significantly (Bhati et al., 2020), and the media played a pivotal role in transmitting information and shaping people’s risk perceptions.

Central to understanding the behavioral shifts in the face of such a health threat is the Protective Motivation Theory (PMT) (Wurtele and Maddux, 1987; Hansen et al., 2023), which postulates that individuals are likely to adopt preventive measures when perceiving potential threats (Wang et al., 2019; Kim et al., 2022; Papagiannidis et al., 2022). Increased risk perception during the pandemic has notably motivated protective behaviors in individuals, particularly those with a higher propensity for risk avoidance (Chua et al., 2021). In practice, people have adopted protective travel behaviors, including reducing work travel, stopping travel to moderate/high-risk destinations, and favoring short/medium-distance domestic locations for leisure travel (Hotle et al., 2020; Agag et al., 2023).

Amid the initial outbreak, extensive media coverage contributed significantly to public concerns and fear, prompting protective behaviors against infection (Savadori and Lauriola, 2021). Most people rely on specific types of media for information about public hazards (Tomczyk et al., 2022). According to Media Effect Theory (MET), the manner in which media communicates this information can shape an individual’s risk assessment, which, in turn, influences their intentions to adopt protective measures (Savadori and Lauriola, 2021). Media Use Theory (MUT) tells us that personal exposure to media also impacts risk perception and intentions to adopt protective behaviors (Oh et al., 2021).

Media consumption has dramatically evolved with the advent of new technologies (Salman et al., 2011). The term “new media” -- contrasting with “traditional media” like newspapers, books, and TV broadcasts -- refers to interactive, user-generated digital content (Bezjian-Avery et al., 1998; Flew, 2008; Woo et al., 2014; Siapera, 2018). Although definitions vary, for this study, we define new media as a network medium that uses digital technologies and the internet to disseminate, diffuse, and exchange information. It’s noteworthy that both new and traditional media influence cognition and behavior, but their impacts vary in degree and intensity (Hennig-Thurau et al., 2010; Wu and Li, 2017).

Previous studies have considered how COVID-19 impacted tourist behavior, the evolution of potential tourists’ risk perceptions during an outbreak, and how media exposure influences these perceptions (Yang et al., 2022b). Nevertheless, there is insufficient research on how different media sources shape travel-related protective behavior intentions and the role played by risk perception in this context. While an array of plausible research examines pairwise relationship between media use, risk perception, and behavioral intentions (Bhati et al., 2020; Neuburger and Egger, 2020), investigations into the varied effects of new and traditional media on travel risk perceptions and travel protective behavioral intentions during an epidemic remain absent.

Therefore, this research aims to fill this gap by exploring how new and traditional media influence Chinese consumers’ risk perception and their subsequent travel-related protective behaviors. This objective will be achieved by constructing a model depicting the influence of media use on risk perception and travel protective behavioral intentions during COVID-19. The findings from this study seek to offer imperative and timely recommendations to tourism industry operators regarding the implications of diverse media use for post-pandemic marketing strategies.

## 2. Literature review

### 2.1. Media use intensity and risk perception

Media use theory is applicable for analyzing the motivations, frequency, duration, and effects of media use on individuals’ behaviors and opinion formation (Schneiders et al., 2022). On the other hand, Media effects theory is suitable for studying the direct or indirect influences of media on individuals’ cognition, attitudes, and behaviors, encompassing various subfields such as social cognitive theory, selective perception, and motivation (Valkenburg et al., 2016). Media plays a significant role in shaping an individual’s risk perception by providing information about potential risks and strategies to mitigate them (Frh, 2017). Risk perception is individuals’ subjective judgments of the likelihood of negative events that may pose immediate or long-term threats to their health and well-being (Johnson et al., 2023). When the perceived risk exceeds their acceptable threshold, individuals may adapt their travel plans accordingly (Airak et al., 2023).

Media exposure and social influence are external factors that often influence risk perception (Liu M. et al., 2020). During the COVID-19 pandemic, the public relies on various types of mass media for information about COVID-19 and the associated travel risks (Ma et al., 2021). Consequently, different types of media contribute to the formation of individuals’ risk perception (Du et al., 2021). For example, exposure to COVID-19 information from mainstream media sources such as cable news channels, local news channels, and newspapers increases individuals’ perceived vulnerability (Olagoke et al., 2020).

The use of social media platforms is associated with higher levels of psychological and social anxiety regarding potential risks, consequently leading to increased risk perception. Moreover, research indicates that the use of new media (both official and unofficial sources) affects the public’s perception of risk (Zhou, 2022). Specifically, frequent exposure to epidemic information through new media platforms is linked to higher risk perception (Li et al., 2021). This risk perception may even surpass that of individuals relying solely on traditional media sources (Huang et al., 2018). Therefore, this study hypothesizes as follows:

*H1:* The higher the intensity of new media use, the stronger the COVID-19 risk perception.

*H2:* The higher the intensity of traditional media usage, the stronger the COVID-19 risk perception.

### 2.2. Media use intensity and tourist protection behavior intention

Both media effects theory and social amplification theory of risk propose a relationship between media usage and behavior (Pidgeon, 2010). Under risk events, individuals perceive risks transmitted by the media and this affects their behavior (Wu and Li, 2017). As a result, the media plays a crucial role in promoting preventive behavior, amplifying the epidemic messages from the traditional media, and thus preventing large infection outbreaks. Social media provides the same effect of encouraging individuals to take preventive behaviors during a crisis (Seo, 2019). Furthermore, both threat and coping appraisals of public health events can enhance travelers’ protection motivations, which in turn affect their actual behaviors (Wang et al., 2019). Here tourism protection behavior intention is defined as the subjective tendency of individuals to take actions to protect, promote, or maintain their health and safety in the context of an epidemic. Amplification of official messages through media channels increases the likelihood that travelers will protect themselves against health risks. Previous research indicates that media use affects travel behavior intention. For example, the intensity of social media use affects tourists’ cognitive image of a destination and travel behavior intention (Huang et al., 2018). On the other hand, heavy Internet use is associated with reduced travel away from home (Lachapelle and Jean-Germain, 2019). Media usage can also influence adoption of environmentally friendly travel behaviors, with various types of media having different degrees of influence (Borowski et al., 2020). The effect of media exposure on preventive behavioral intentions has been proposed as due to it influencing subjective norms (Liu L. et al., 2020). The more epidemic information tourists are exposed to, the stronger effect on tourism behavioral intention (Cahyanto and Liu-Lastres, 2020). Therefore, this paper proposes the following hypothesis:

*H3:* New media use intensity positively affects travel protective behavior intention.

*H4:* Traditional media use intensity positively affects travel protective behavior intention.

### 2.3. Epidemic risk perception and tourist protective behavioral intention

Protection motivation theory considers that people’s risk perception is a key factor affecting behavioral intentions (Floyd et al., 2000). Empirical studies have found that people decide whether to take protective behaviors depending on their perception of the risk situation (Kowalski and Black, 2021; Nazione et al., 2021). A study of the Ebola virus shows that the higher a person’s perceived susceptibility, severity and travel risk, the more likely they are to avoid travel (Cahyanto et al., 2016). Tourists with a higher perception of the risk of smog haze are likely to change their travel plans based on the weather conditions and their effect on haze levels (Wu and Li, 2017). Similarly for COVID 19, the higher the individual perceives their susceptibility to the coronavirus, the greater their intention to take protective travel behavior (Raina et al., 2022), by changing their travel patterns or reducing travel frequency (Abdelrahman, 2022). The stronger the risk consequence, the lower the travel intention of tourists (Cahyanto and Liu-Lastres, 2020).

While Protection Motivation Theory (PMT) includes efficacy beliefs, this study’s primary focus is on risk perception for several reasons. In high-risk scenarios like pandemics, risk perception often supersedes efficacy beliefs in shaping behavior (Bish and Michie, 2010; Dryhurst et al., 2020). The urgency and immediacy of the COVID-19 threat made risk perception an apt focal point (Siegrist et al., 2021). Furthermore, focusing on risk perception streamlines our hypothesis testing, providing a targeted understanding of the situation. This focus is crucial in time-sensitive cases like pandemics, as demonstrated during the influenza A (H1N1) outbreak where risk perception significantly impacted protective measure adoption (Weerd et al., 2011). However, we recognize that efficacy beliefs, alongside trust and risk perception, form an important aspect of PMT, shaping behavioral outcomes (Siegrist et al., 2021). Our study has not incorporated this perspective, but it provides an enriching opportunity for future research in this domain. Therefore, in light of these findings, this paper posits the following hypothesis:

*H5:* The higher epidemic risk perception, the greater travel protective behavior intention.

### 2.4. The mediating effect of risk perception on the relationship between media use and travel protection behavioral intentions

An individuals’ interpretation of information and events will directly affect whether they act (Albarracín and Wyer, 2000; Ma and Cao, 2019). Related media psychology research has found that individuals who use media can either be harmed or helped, depending on their personal cognition, which acts as a bridge between external information and behavioral consequences (Stever et al., 2021). Previous tourism research has found that risk perception plays a mediating role between media use and tourism behaviors. However, the intermediary role of risk perception on the relationship between media use and tourism behavior has not been examined (Chien et al., 2016). Instead, studies have focused on the relationship between destination image and the epidemic situation. For example, destination image perception plays a complete intermediary role between social media user generated content and tourism intention (Huang et al., 2018), and personal perception plays a mediating role between epidemic situation and protective behavior (Luo and Wang, 2021), or tourism behavior intention (Lebrun et al., 2021). Other studies have examined how media affects individual health attitudes and encourages individuals to adopt protective behaviors (Giustini et al., 2018). Therefore, this paper proposes the following hypotheses:

*H6:* COVID-19 risk perception has a mediating effect between new media use intensity and travel protective behavior intention.

*H7:* COVID-19 risk perception has a mediating effect between traditional media use intensity and travel protective behavior intention.

Section 2.3 of our study explores why risk perception, rather than efficacy beliefs, is more crucial during high-risk crises like pandemics. Hence, our research narrows down its focus to the impact of media use intensity on tourism behavior in the COVID-19 pandemic, emphasizing risk perception. Efficacy beliefs, although noteworthy, are not included in the current study. Future research might explore this aspect, but the current research primarily probes risk perception’s influence. This approach is based on our premise that high-risk crises make risk perception a more pressing and influential factor in shaping behaviors.

### 2.5. The conceptual model

This study aims to understand how media use affects behavioral decision-making during the COVID-19 pandemic, taking into account the theoretical perspectives of the Media Use Theory (MUT), Media Effect Theory (MET), Social Amplification Theory (SAT), and Protection Motivation Theory (PMT). These integrated theories provide a rigorous theoretical foundation for the conceptual model and enhance the analysis of the relationships between media use, risk perception, and travel protection behavior intentions. The revised conceptual model hypothesizes that an individual’s exposure to epidemic information in the media, influenced by the Social Amplification Theory, Media Setting Theory, and Protection Motivation Theory, directly affects their risk perception and travel protection behavior intentions and can also indirectly affect travel protection behavior intentions through the mediation of risk perception.

**Figure 1:**
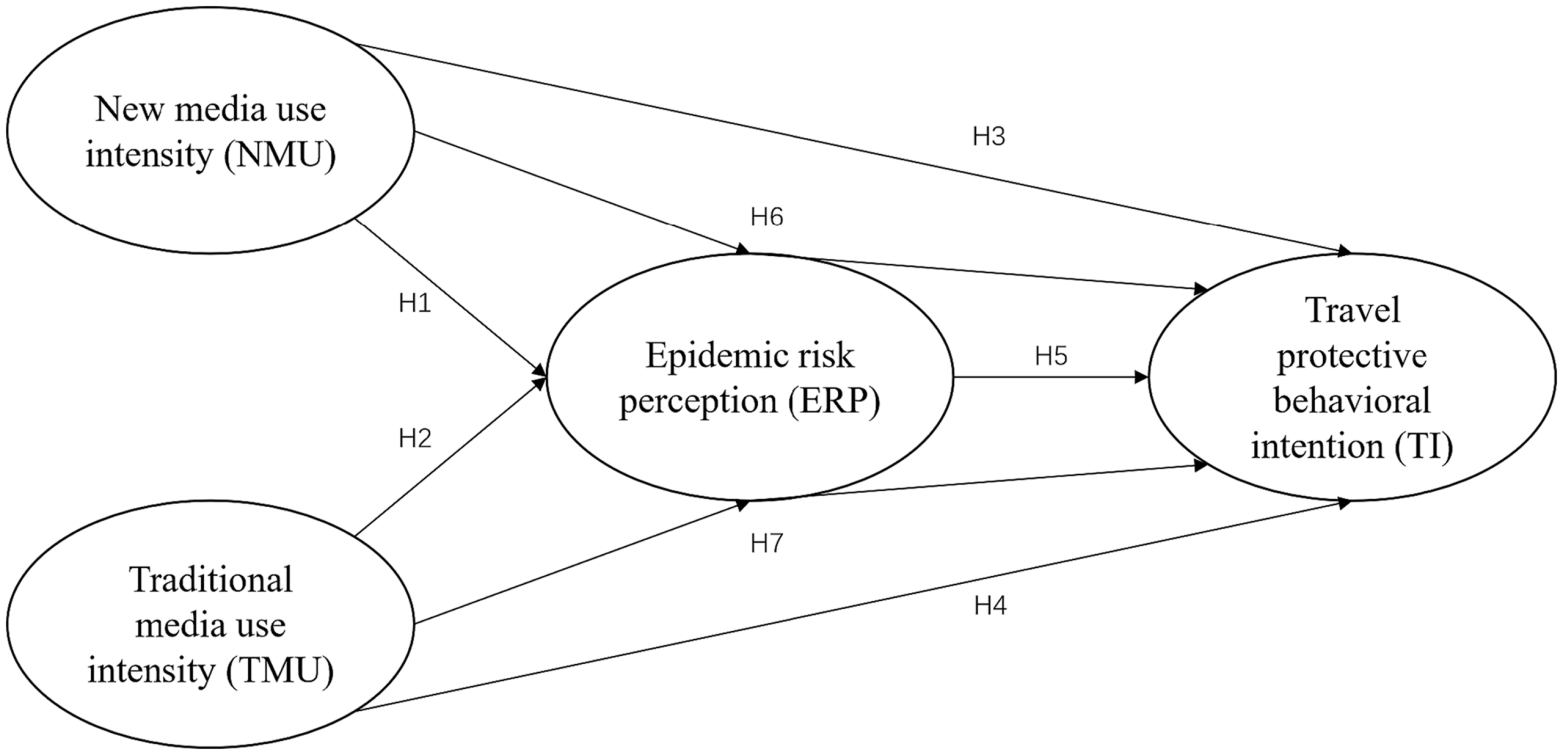
Conceptual model of the influence of media use intensity on epidemic risk perception and travel protective behavioral intention.

In this revised model, the Media Effect Theory emphasizes how media exposure shapes an individual’s risk perception by providing information about potential risks and how to avoid them. The Social Amplification Theory suggests that media usage can amplify the transmission of risk messages and influence individuals’ behavior during risk events. The Protection Motivation Theory considers the role of risk perception in influencing individuals’ behavioral intentions and decision-making in response to threats. By incorporating these integrated theories into the conceptual model, the study establishes a rigorous theoretical foundation that justifies the continued relevance of the research (see Figure 1).

## 3. Methodology

The present study is based on empirical data collected through a survey to test the hypotheses proposed in the theoretical model.

### 3.1. Research design and variables choosing

The questionnaire consists of two main parts. The first collects the demographic characteristics of the respondents, including gender, age, education level, occupation, and monthly disposable income. The second contains the measurement items of the variables in the research model. By design, the measurement instrument drew upon scales used in past research to ensure the instrument’s consistency, reliability, and validity. The measures were adapted to the context of the analysis, which was the pandemic situation. The model includes four latent variables. The intensity of media use means the extent to which a person is actively engaged in different media supported activities. The article examines the previous measurement standards and analysis indicators of media intensity (Rosen et al., 2013; Du et al., 2021; Oh et al., 2021). To understand the degree of dependence of the respondents on different media under the condition of multiple media exposure, this paper has designed indicators of new media use intensity and traditional media use intensity. This article explores the research on how people perceive the risks of an epidemic (Bae and Chang, 2020; Sánchez-Cañizares et al., 2021), and understand the views of the respondents in terms of the severity of the epidemic itself, and the susceptibility and severity of infection sequelae. Seven dimensions for measuring protective tourism behavior are proposed (Wang et al., 2019; Neuburger and Egger, 2020; Lebrun et al., 2021). The specific items are all measured using a 5-point Likert-type scale. The intensity of media use is measured by asking participants the extent that they have been exposed to media information about epidemic risks (1 = not at all, to 5 = to a great extent) (see Table 1).

**Table 1:** Latent variables and measured variables.

Latent variable	Measurement questions	Reference
New media use intensity (NMU)	NMU_1_: The degree to which you have received information related to the epidemic from social media (such as Weibo, WeChat, Tiktok, Zhihu, Bilibili, etc.)	Rosen et al. (2013), Du et al. (2021), Oh et al. (2021), etc.
	NMU_2_: The degree to which you have received information about the epidemic from current news websites/news clients (such as Sina.com, Xinhua.net, Tou Tiao, etc.)	
	NMU_3_: The degree to which you have received information about the epidemic from portals/forums (such as Tencent, Net Ease, Sohu, etc.)	
Traditional media use intensity (TMU)	TMU_1_: The extent to which you have received information about the epidemic from TV	Liu L. et al. (2020), Vai et al. (2020)
	TMU_2_: The extent to which you have received information about the epidemic from newspapers/magazines	
	TMU_3_: The extent to which you have received information about the outbreak from medical newsletters or journals	
	TMU_4_: The extent to which you have received information about the epidemic from the broadcast radio	
Epidemic risk perception (ERP)	ERP_1_: The new coronavirus is a very serious disease	Bae and Chang (2020), Chua et al. (2021), Sánchez-Cañizares et al. (2021)
	ERP_2_: Humans are highly susceptible to the new coronavirus	
	ERP_3_: Infection by the new coronavirus seriously harm to the body	
Travel protection behavioral intention (TI)	TI_1_: I will avoid traveling during the holidays	Wang et al. (2019), Neuburger and Egger (2020), Lebrun et al. (2021)
	TI_2_: I will shorten the travel time	
	TI_3_: I will travel less frequently	
	TI_4_: I will choose the surrounding cities to travel	
	TI_5_: I will choose domestic travel	
	TI_6_: I will choose areas where there is no epidemic to travel	
	TI_7_: I would rather spend more money to ensure the safety of travel	
	TI_8_: I intend to travel after the epidemic is over	
	TI_9_: I intend to travel after vaccination	

NMU represents “new media use intensity”; TMU represents “traditional media use intensity”; ERP represents “Epidemic risk perception”; TI represents “travel protection behavior intention”.

### 3.2. Data sample

Data was collected from the residents of Shanghai Songjiang University Town. Songjiang University Town residents have the highest trips per year in Shanghai. The residents therefore will be expected to have been strongly affected by the COVID-19 lockdowns. Before the formal survey, the researcher conducted a trial survey (*N* = 76) in early June 2021. After removing invalid questionnaires, the researcher analyzed the reliability and validity of 71 valid questionnaires and deleted and revised the problematic items. The distribution and collection of formal questionnaires were carried out in late July and August of 2021. In July 2021, Shanghai reported a total of 108 confirmed cases of COVID-19, all of which were imported cases. In August 2021, Shanghai reported a total of 171 confirmed cases of COVID-19, including 10 local cases and 161 imported cases. In late July 2021, the Shanghai Culture and Tourism Administration issued a notice on strict implementation of epidemic prevention and control measures. On August 6, Shanghai suspended trans-provincial group tours (SMACT, 2021). The questionnaires were distributed online and collected by snowballing. A total of 650 online questionnaires were distributed and 594 were returned. A consistency test of the samples was carried out. A total of 103 invalid questionnaires were excluded due to blanks and omissions or to most questions on a response being the same number. A total of 491 valid questionnaires were retained (82.66%).

The sample consisted of 43.6% male and 56.4% female respondents, which is in line with the local area gender characteristics. In the sample, those 18–25 years old accounted for 41.1% of the total and 25–50 years old respondents accounted for 42.4%. Most of the participants have a higher education level; 59.1% of the participants are college/undergraduate students, 22.4% have a master’s degree or above. Monthly disposable income was found in the following ranges: less than 2,480 Rmb accounting for 40.5%, 2,481–5,000 Rmb accounting for 18.1%, and 5,001–10,000 Rmb accounting for 33.2%. The occupations of the participants were mainly students accounting for 39.5%. The sample population’s educational attainment, income level, and other status variables are associated with the higher proportion of students in the sample area, as well as the demographic characteristics of the university town (Table 2).

**Table 2:** Respondent key demographic variables.

Variable	Frequency	%	Valid %	Cumulative %
*Gender*				
Female	277	56.4	56.4	56.4
Male	214	43.6	43.6	100
*Age*				
< 18	13	2.6	2.6	2.6
< 25, > = 18	202	41.1	41.1	43.7
< 50, > = 25	208	42.4	42.4	86.1
> = 50	81	13.9	13.9	100
*Occupation*				
Student	194	39.5	39.5	39.5
Government and public institutions	37	7.5	7.5	47
Enterprise	176	35.8	35.8	82.9
Others	84	17.1	17.1	100
*Education*				
Junior high school and below	34	6.9	6.9	6.9
High school or technical secondary school	57	11.6	11.6	18.5
College and undergraduate	290	59.1	59.1	77.6
Master or above	110	22.4	22.4	100
*Income (Rmb Yuan/month)*				
< 2,480以下	199	40.5	40.5	40.5
2,480–5,000	89	18.1	18.1	58.7
5,000–10,000	163	33.2	33.2	91.9
> 10,000	40	8.1	8.1	100
*Have you ever traveled*				
Yes	435	88.6	88.6	88.6
No	56	11.4	11.4	100
*Total*	491	100	100	

Given the youthful demographic of the University Town, we surmise that the influence of new media was potentially stronger in this sample. Nevertheless, it is crucial to note the increasing universality of smart device use. As of 2021, there were 950 million smartphone users in China, an adoption rate of 68%. Almost all eligible age groups in Shanghai had widespread smartphone access. Particularly, Shanghai statistics indicate that 88.1% of residents aged 60 and above own a smartphone. Consequently, the likelihood of information acquisition *via* new media is growing across all age groups. Research by Deloitte in 2022 further corroborates this when it revealed increasing smartphone dependency across all age groups during the COVID-19 pandemic. Truthfully, younger populations might have more exposure to new media, but older populations are also trending in this direction.

Emphasizing the sample’s affiliation to Songjiang University Town - an up-and-coming, technologically advanced area in Shanghai - underscores the potential futuristic relevance of the findings. Despite a presently limited representation, particularly from seniors and individuals below 18, the research offers valuable insights into travel decisions that may indeed shape those of future Chinese populations. This younger demographic, with their strong reliance on new media and rational decision making, could potentially form a model for examining and predicting travel behaviors and risk perceptions in the rapidly evolving technology and information landscape.

## 4. Results

Data were analyzed using SPSS22.0 and AMOS24.0 software. The initial phase of the analysis involved undertaking an Exploratory Factor Analysis (EFA) through SPSS 22.0, with the objective of discerning the underlying structure of the data and identifying distinct factors. Subsequent to this EFA, a Confirmatory Factor Analysis (CFA) was conducted using AMOS 24.0 to validate and confirm the factor structure elicited from the exploratory phase. Following these preliminary analyses, Structural Equation Modeling (SEM) was utilized to scrutinize the structural model and examine the hypothesized relationships within the model. In the final stage of our analysis, we deployed Multi-Group Analysis (MGA) to test the robustness and applicability of our model across different demographic groups.

### 4.1. Scale reliability and validity test

Exploratory factor analysis was used to test the reliability and validity of the questionnaire variables. It is important to note that the EFA was carried out using data derived specifically from the trial survey, with a sample size of 76 participants. Cronbach coefficient is the most used measure of reliability. Cronbach’s alpha values of the four variables are 0.798, 0.845, 0.814, 0.846, above the acceptable level of 0.7. The Kaiser-Meyer Olkin (KMO) test and Bartlett test were used to find if the data were suitable for factor analysis. The KMO of the four variables are 0.712, 0.787, 0.707, 0.910 respectively, and the *p* value of the four variables is 0.000, KMO > 0.7, *p* < 0.05, indicated the adequacy of the sample size as well as the existence of the latent variables. The KMO value for the total scale items is 0.876, and Bartlett’s test of sphericity (*χ*^2^ = 3,165.854, *p* = 0.000) is significant, indicating that the scale is appropriate for factor analysis. Exploratory factor analysis of the scale items was carried out using principal component analysis. Four factors with a factor load greater than 0.5 were extracted. The result after rotation shows that the loading of TI2 was less than 0.5 and hence it was deleted. After deleting this item, factor analysis was carried out again, and five factors were extracted with factor loadings greater than 0.5, and the cumulative explained variance was 62.985%. This indicates that the internal consistency of scale structure was valid, and the screened factors are representative.

To further investigate the validity of the scale, this paper uses AMOS24.0 software to conduct confirmatory factor analysis to test the relationship between observed variables and latent variables. Notably, the CFA was undertaken utilizing a comprehensive dataset derived from the total survey, which included a sample size of 491 participants. First the convergent validity of the scale the discriminant validity among variables was tested. Table 3 shows the results of Convergent validity test analysis. Convergent validity is usually measured with CR and AVE. If CR > 0.7 and AVE > 0.5, it indicates that the aggregation validity is good. If AVE < 0.4, the variables need to be deleted to improve the convergence effect. The result shows that the AVE of tourism protection behavior intention is only 0.395, and the loadings of TI7 and TI6 are low, so they should be deleted. After deleting the items, the AVE value of tourism behavior intention is 0.402. This AVE value is less than 0.5 but greater than 0.4 due to the sample size, so it is within the acceptable range. Overall, the convergence validity of the scale is high. Table 3 shows the results of the reliability and conversion validity test analysis.

**Table 3:** Reliability and validity test of each variable measurement scale.

Factors and items	Estimate	Standard error	*T* value	*p* value	Cronbach’s α	CR	AVE
New media use intensity					0.798	0.801	0.573
NMU1	0.740						
NMU2	0.754	0.083	14.286	^***^			
NMU3	0.776	0.078	14.486	^***^			
Traditional media use intensity					0.845	0.847	0.583
TMU4	0.660						
TMU3	0.836	0.086	14.848	^***^			
TMU2	0.780	0.080	14.244	^***^			
TMU1	0.767	0.081	14.076	^***^			
Epidemic risk perception					0.814	0.817	0.598
ERP1	0.827						
ERP2	0.729	0.053	15.299	^***^			
ERP3	0.761	0.056	15.793	^***^			
Travel protective behavior intention					0.830	0.801	0.402
TI1	0.719						
TI3	0.664	0.073	12.796	^***^			
TI4	0.613	0.074	11.918	^***^			
TI5	0.641	0.075	12.397	^***^			
TI8	0.594	0.079	11.568	^***^			
TI9	0.561	0.073	10.983	^***^			

CR means composite reliability; AVE means average variance extracted.

Discriminant validity is used to test the difference between two factor constructs (Iacobucci, 2010). Discriminant validity was tested by comparing the square root of AVE for individual constructs with the correlations among the latent variables. As shown in Table 4, the square root of AVE exceeded those of the off-diagonal elements, which means the measurement scale of each latent variable has good discriminant validity.

**Table 4:** Differential validity test of latent variables.

Dimension	Travel protective behavior intention	Epidemic risk perception	Traditional media use intensity	New media use intensity
Travel protective behavior intention	0.573			
Epidemic risk perception	0.373	0.583		
Traditional media use intensity	0.307	−0.156	0.598	
New media use intensity	0.462	0.053	0.529	0.402
SQRT(AVE)	0.757	0.764	0.773	0.634

SQRT (AVE) means the square root of AVE.

In a confirmatory factor analysis, model fit refers to how closely observed data match the relationships specified in a hypothesized model (Ribeiro et al., 2017). The commonly used fit indexes include two judgment criteria: the similarity index and dissimilarity index between the two models. The similarity index includes the goodness-of-fit index (GFI), adjusted goodness-of-fit index (AGFI), comparative fit index (CFI), and Tucker-Lewis index (TLI). Dissimilarity indicators include the root mean square error of approximation (RMSEA) and the standardized root mean square residual (SRMR). As shown in Table 5, the model has a good degree of fit since it meets critical value requirements of each index.

**Table 5:** Model fit test.

Fit metrics	*χ*^2^/degrees of freedom	SRMR	RMSEA	GFI	AGFI	IFI	CFI	TLI
Test value	2.988	0.058	0.064	0.928	0.900	0.932	0.933	0.918
Reference	<3.000	<0.080	<0.080	>0.090	>0.090	>0.090	>0.090	>0.090

SRMR means standardized root mean square residual; RMSEA means root mean square error of approximation; GFI means goodness-of-fit index; AGFI means adjusted goodness-of-fit index; IFI means incremental fit index; CFI means comparative fit index; TLI means Tucker-Lewis index.

### 4.2. Path analysis and hypothesis testing

After the reliability and validity of the scales meet the requirements, the path analysis is used to measure the relationships between the independent and dependent variables to verify whether the hypotheses are established. AMOS24.0 software is used to analyze the path of the model and the following results are obtained.

**Figure 2:**
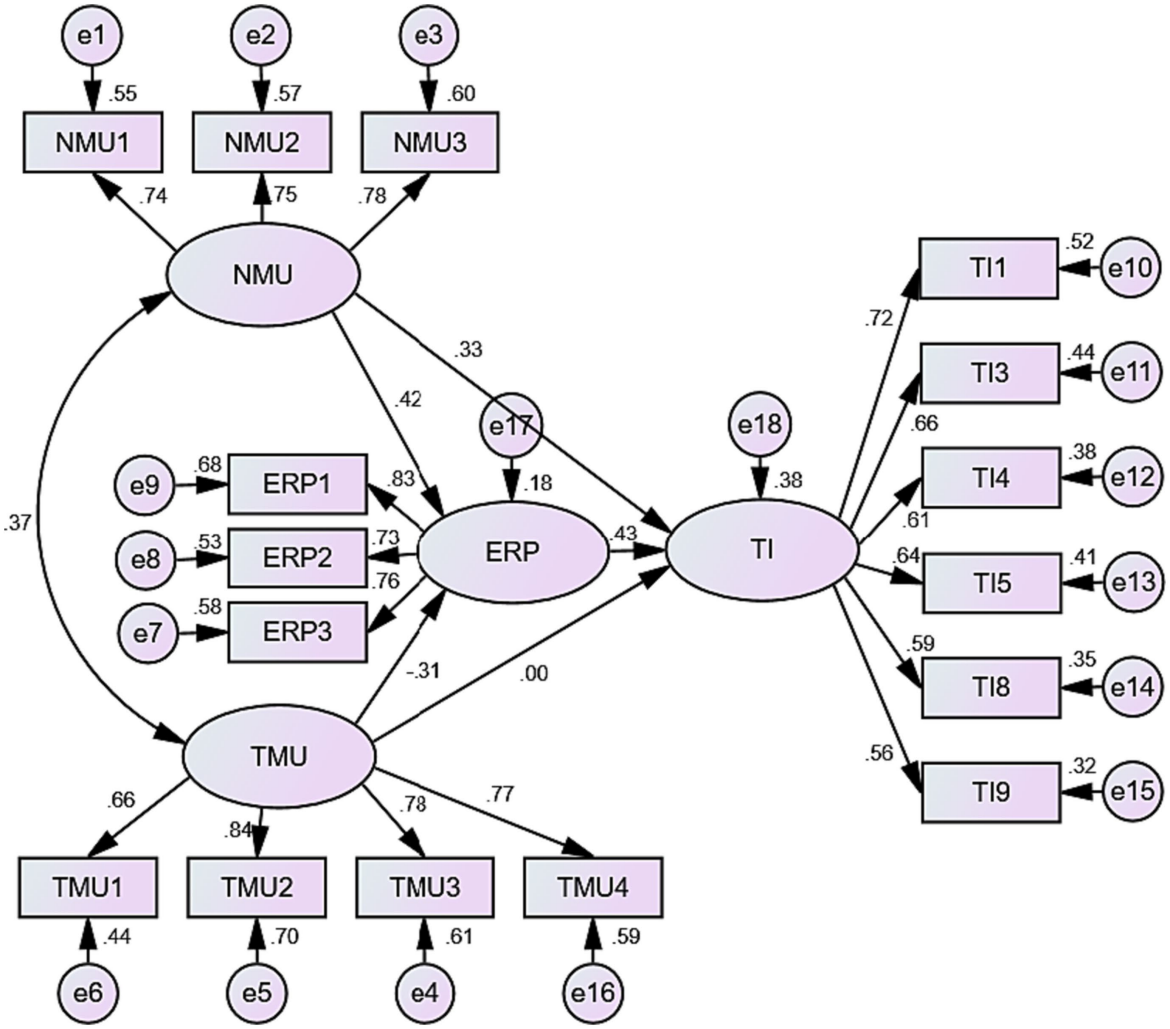
Structural equation model diagram and path analysis.

As shown in Figure 2, the intensity of new media use is significantly associated with both epidemic risk perception and tourism protection behavior intention. However, the intensity of traditional media use has a significant negative impact on the perception of epidemic risk but has little correlation with the behavioral intention of tourism protection. The results of this research suggest that individuals who perceive a higher risk of an epidemic are more likely to have a stronger intention to engage in protective behaviors concerning tourism.

Table 6 provides the results of the path analysis and hypotheses verification. The path analysis showed that, except for the insignificant effect of traditional media use intensity on tourism protection behavior intention, the other variables were all significantly related. The data does not support H4 but does support the other hypotheses.

**Table 6:** Model path analysis and hypothesis test results.

Hypothesis	Path relationship	S.E.	C.R.	*p*	Estimate	Test
H1	NMU → ERP	0.070	6.617	^***^	0.424	True
H2	TMU → ERP	0.048	−5.260	^***^	−0.315	True
H3	NMU → TI	0.051	5.123	^***^	0.333	True
H4	TMU → TI	0.033	−0.087	0.931	−0.005	False
H5	ERP → TI	0.045	6.881	^***^	0.426	True

Estimate represents standardized estimate; S.E. represents standard error of estimated parameters. C.R. represents critical ratio. * means *p* < 0.1, ** means *p* < 0 0.05, *** means *p* < 0.01.

### 4.3. Total effect, direct effect and indirect effect

This paper examines whether epidemic risk perception mediates the relationship between media use intensity and tourism protection behavior intention, by estimating the total, direct, and indirect effects. The results of the effect analysis conducted using AMOS24.0, are presented in Table 7. The total effect of an independent variable on another dependent variable is the sum of its direct effect and indirect effects. The total effect of traditional media use intensity on tourism protection behavior intention is −0.139, of which the direct effect is −0.005, while the indirect effect through epidemic risk perception is −0.134. This means the total effect of traditional media use intensity on tourism protection behavior intention is significant, and the negative relationship is mediated by epidemic risk perception. H7 is supported.

**Table 7:** Effect test.

Standardized total effects			
	TMU	NMU	ERP
ERP	−0.315	0.424	0.000
TI	−0.139	0.514	0.426
Standardized direct effects			
	TMU	NMU	ERP
ERP	−0.315	0.424	0.000
TI	−0.005	0.333	0.426
Standardized indirect effects			
	TMU	NMU	ERP
ERP	0.000	0.000	0.000
TI	−0.134 (**H7 supported**)	0.181 (**H6 supported**)	0.000

−0.134 (H7 supported) represents an indirect effect of −0.134 between TI and NMU, supporting hypothesis H7. 0.181 (H6 supported) represents an indirect effect of 0.181 between TI and ERP, supporting hypothesis H6.

The total effect of new media use intensity on tourism protection behavior intention is 0.514, of which the direct effect is 0.333, while the indirect effect through epidemic risk perception is 0.181. Epidemic risk perception partially explains the relationship between new media use intensity and tourism protection behavior intention. H6 is supported.

### 4.4. Multi-group analysis (MGA) based on demographic variables

Multi-Group Analysis (MGA) is a statistical method used in structural equation modeling (SEM) when research involves comparison between two or more groups (Li et al., 2023). This approach facilitates the examination on whether construct relationships, as theorized in our model, hold uniformly across various groups. Notable variations emerge based on different demographic factors. Gender variations, for instance, have distinct behavioral intentions (Yang et al., 2022c). Similarly, age or generational groups exhibit unique intentions (Wu et al., 2023), while geographic aspects also contribute to travel behavior (Yang et al., 2022a). These analyses offer insights into demographic influences on the relationship among media use intensity, travel risk, and behavioral intentions.

Table 8 represents the model fit test for the multiple group analysis (MGA) conducted on different demographic variables. The significant Chi-square values suggest that the demographic variables in the model have an influence on the relationship between media use intensity, risk perception, and travel protective behavior intention. The good fit of the model further supports the validity of the MGA in analyzing these relationships.

**Table 8:** Model fit of MGA.

Model fit	Chi-square	Degrees of freedom	Probability level	CMIN/DF
Gender	404.883	196	***	2.066
Occupation	342.885	196	***	1.749
Age	430.128	196	***	2.195
Education	383.981	196	***	1.959
Income	383.682	196	***	1.958
With or without travel experience	434.273	196	***	2.216

* means *p* < 0.1, ** means *p* < 0.05, *** means *p* < 0.01.

#### 4.4.1. Gender differences

No significant gender differences were observed in media use intensity, perceived travel risk, and travel protective behavioral intentions. Traditional media use intensity was negatively correlated with perceived travel risk for both males and females. New media use intensity positively influenced both risk perception and protective behavioral intentions for both genders. Risk perception had a significant positive impact on travel protective behavior for both males and females.

#### 4.4.2. Education level differences

Across all education levels, there was a positive relationship between new media use intensity and risk perception, as well as travel protective behavior intentions. Traditional media use intensity was negatively correlated with perceived travel risk across all education groups. However, its influence on travel protective behavior intentions was significant only for individuals with a high school or vocational education level. Risk perception had a positive impact on behavior intentions for individuals in other education groups.

#### 4.4.3. Income groups

For all income groups, new media use intensity positively influenced perceived risk and travel protective behavior intentions. Traditional media use intensity was negatively correlated with perceived travel risk across all income groups. The influence of traditional media use intensity on travel protective behavior intentions varied across different income groups.

#### 4.4.4. Age groups

Across all age groups, new media use intensity positively influenced travel protective behavior intentions. With the exception of individuals aged 50 and above, there was a positive correlation between new media use intensity and perceived risk for all other age groups. Traditional media use intensity was negatively correlated with perceived travel risk for all age groups. However, the relationship between traditional media use intensity and travel protective behavior intentions varied across age groups.

#### 4.4.5. Occupation groups

The impact of traditional media use intensity on epidemic risk perception and travel protective behavior intention is consistent across different occupational groups. Traditional media use intensity is negatively correlated with epidemic risk perception in all occupational groups but does not significantly affect travel protective behavior intention in these groups. Conversely, new media use intensity is significantly and positively correlated with travel protective behavior intention across all occupational groups. Except for the Government and Public Institutions group, the relationship between new media use intensity and epidemic risk perception, as well as the relationship between epidemic risk perception and travel protective behavior intention, follows consistent patterns across the other groups, with significant positive correlations.

#### 4.4.6. With or without travel experience

Within both groups, individuals with and without travel experience exhibited a similar pattern of relationships between the variables. Specifically, new media use intensity positively influenced risk perception and travel protective behavior intentions, whereas traditional media use intensity was negatively correlated with risk perception. However, in both groups, no significant relationship was found between traditional media use intensity and travel protective behavior intentions.

## 5. Discussion

### 5.1. Interpretation of results

Our investigation revealed a significant positive association between new media use intensity and both epidemic risk perception, as well as an intention to adopt safety-conscious tourism behaviors. This is consistent with other scholars’ research on the influence of social media on perception (Huang et al., 2018; Liu M. et al., 2020), and also in line with research about the impact of social media on tourism intentions (Koo et al., 2016; Yoo et al., 2016). This suggests that as individuals’ new media usage escalates, their perceived sense of epidemic risk intensifies, which in turn has the potential to spark an increased intent to enact more vigilant tourism behaviors (Pakpour et al., 2021; Xie, 2021; Jiang et al., 2022).

Yet, our exploration into the role of traditional media use yielded contrasting results. We found a direct inverse association between traditional media usage and risk perception, but surprisingly, there wasn’t any marked influence on protective behavior. While this finding resonates with some previous studies (Liu L. et al., 2020), it contradicts others (Luo and Wang, 2021), exhibiting the contentious nature of traditional media’s influence on public risk perception.

These disparities might be attributed to several factors. For instance, the level of pandemic control, the promptness of epidemic information release, and the effectiveness of prevention measures (Liu M. et al., 2020). All these elements influence the impact traditional media use has on risk perception. The contradictory results underscore the need for further research to create a more nuanced understanding of traditional media’s role in shaping perceptions and behaviors during epidemics (Popiołek et al., 2021).

Upon examination of different demographics, we found that the influence of media usage and risk perception varies across age, education level, and occupation, which corroborates with what Salman et al. (2011) found. However, a novel finding in our study was that government and public institution employees displayed lower perception of COVID-19 severity and susceptibility, which is not commonly reported in existing literature. We also identified the influence of income level on media usage patterns, which is an underexplored area.

These mixed results highlight the complexity of media influences, suggesting that the impact of media on different population segments is not uniform. This is an important detail that is often overlooked in current research. Our study contributes to a more comprehensive understanding of the multifaceted impacts of media during health crises, adding depth to the current knowledge landscape.

### 5.2. Implications of the findings

Our study provides meaningful conclusions, sketching an assertive picture of the critical role media, both new and traditional, play in molding public perception and subsequent actions during an epidemic. These platforms carry a substantial responsibility to transmit reliable and accurate information to the public, a role that has been amplified in our findings.

The differing influences of traditional and new media indicate that an effective epidemic communication strategy should not be a one-size-fits-all approach. Rather, it necessitates a finely-tuned balance, fully leveraging the strengths and acknowledging the weaknesses of each media type. For instance, taking into account our findings, public health officials could utilize new media as a tool to enhance risk perception and safety-oriented behaviors, owing to its positive correlation with these aspects. Simultaneously, the role of traditional media in managing lower risk perceptions should not be overlooked, hinting at its use as a tool for assuaging public panic during health crises.

Next, a key insight stemming from our study is the highlight of socio-demographic disparities in media usage and their corresponding influences. This indicates a pressing need for segmented and personalized messaging. For instance, strategies should recognize that younger individuals, more likely to use new media, may perceive higher epidemic risks due to this medium’s usage. On the other hand, older individuals, who lean towards traditional media, might require more reassurances, given the negative correlation between traditional media usage and risk perception. Public health officials could consider these media usage patterns and perceptions while devising segmented communication strategies.

Lastly, our analysis underscores the significant mediating role of perceived epidemic risk in shaping protective behaviors. This finding is particularly invaluable for public health strategists, as it indicates a firm path towards achieving enhanced adherence to safety protocols (Golets et al., 2021). This insight can guide efforts to heighten risk perception through appropriately tailored messaging, thus promoting protective behaviors and helping manage public sentiment both during the current and future health crises.

### 5.3. Suggestions

In response to our findings, we make the following recommendations for enhancing public communication strategies during any health crisis, like an epidemic. These underscore the need for addressing misinformation, enable the leveraging of trusted sources to shape perceptions, and promote suitable behaviors.

Firstly, given the distinct impact of different media types, it is paramount to take into account the specific media platform used for risk communication. Owing to the heightened influence of new media, communication strategies must be directed towards curtailing panic and misinformation that often proliferate on such platforms (Xie and Liu, 2022). Simultaneously, traditional media should be harnessed to disseminate timely, accurate, and soothing information that can reassure and inform the public, hence balancing risk perception.

Secondly, recognizing that media usage and interactions markedly differ among socio-demographic groups, mass communication approaches should be tailored to address these disparities effectively. For instance, targeted messaging and programming could be developed to cater to different age groups, education levels, and occupations. Interventions could also take the form of promoting media literacy initiatives to help the public better decipher and judge the credibility, reliability and salience of the media content they consume (Akritidis et al., 2022).

In addition, collaborations with influencers on new media could be pursued to reach and engage with younger cohorts more effectively. This can establish a more robust public rapport and a more receptive audience to health advisories and recommendations (Xue et al., 2021). This approach further emphasizes the need to keep changing communication strategies, as the media landscape is swiftly transforming.

Lastly, our study underlines the crucial role of perceived epidemic risk - this could anchor strategic communication. It would be beneficial to ensure frequent, clear, and open communication about epidemic statistical trends, procedural changes, and progress updates (Wang and Zheng, 2019). By accurately managing this perception, public understanding can be fostered, promoting appropriate response behaviors. It also enables effective preparation for future public health crises by learning from the current experiences.

In conclusion, our study’s findings suggest a vital need for delicate balance and adaptability in public communications during health crises. It urges towards an audience-centric, data-driven, and dynamic approach to manage risk perception effectively and promote protective behaviors.

## 6. Conclusion

We are living in an era of information overload, and the media is constantly shaping our thoughts and behaviors (Arnold et al., 2023). Especially in the Covid-19, the media has a profound influence on how people perceive risks and behave in terms of protection (Vai et al., 2020; Nazione et al., 2021). The contribution of this paper is in clarifying how new media and traditional media use impact people’s perceptions of epidemic risk and their intention to protect themselves while travelling.

The findings of this study demonstrate that the use of new media improves the public’s perception of epidemic risk and significantly increases their willingness to adopt protective measures. Conversely, frequent use of traditional media has a negative effect on the perception of epidemic risk, leading to a decline in travel protective behavioral intention.

Furthermore, this study explores the differences in media usage tendencies and tourism protection behaviors among various demographic groups, providing valuable insights for tourism companies when developing media marketing strategies for specific target segments. Social media platforms such as TikTok, WeChat, and Weibo are the most commonly used sources of information across all groups. Tourism enterprises can leverage the influence of new media to effectively communicate their safety measures and guarantees, assuaging public concerns regarding risks and rebuilding confidence in the tourism industry, particularly for long-distance and transnational travel (Meng et al., 2021).

While the influence of traditional media has declined overall, it still holds significance for specific groups such as high-income individuals, those above 50 years old, and those with lower educational backgrounds. For this reason, tourism enterprises should adopt a diversified media marketing approach to cater to the preferences of different target groups. Traditional media, particularly television, remains effective in reaching the demographic with lower education levels and the older population. Simultaneously, new media should be utilized to provide high-quality tourism information and engage with the higher-income group that seeks information from multiple sources.

Despite the valuable insights provided by this study, it is important to acknowledge its limitations. The research sample was drawn from residents of Songjiang University Town and may not be representative of the general population in China. Future research should consider expanding the scope by including larger sample sizes and conducting comparative studies across different regions. Additionally, this study was conducted within the context of the COVID-19 pandemic in China, and there may be variations in media influence and attitudes towards risk in different countries (Villacé-Molinero et al., 2021). Comparative studies examining how different epidemic policies influence media impact on risk perception and protective behavior would be beneficial (Selem et al., 2023). Further research efforts, including follow-up studies and spatial–temporal comparisons with the inclusion of additional variables, can provide deeper insights into changing sentiments and attitudes.

In summary, this study contributes to the theoretical understanding of media usage, media influence, social amplification, and protective behavioral intention. It also offers practical implications for tourism enterprises and authorities seeking to rebuild trust and revive the industry in a post-pandemic era. Future research directions should explore different scenarios, expand the sample population, and include additional variables such as efficacy to further enhance our comprehension of the complex dynamics between media, risk perception, and protective behaviors.

## Data availability statement

The raw data supporting the conclusions of this article will be made available by the authors, without undue reservation.

## Ethics statement

Ethical review and approval was not required for the study on human participants in accordance with the local legislation and institutional requirements. Written informed consent from the participants was not required to participate in this study in accordance with the national legislation and the institutional requirements.

## Author contributions

RS and XY conceptualized and managed the research, based on an ongoing interest in cruise ship policy. JT and JY undertook collection and analysis of data. NS provided mentorship, reviewing research procedures and methods, revising drafts and submitting the paper. All authors contributed to the article and approved the submitted version.

## Funding

This study was supported by grants from the National Social Science Foundation of China (to XY) (No. 21BGL281)/(to RS) (No. 22FGLB040).

## Conflict of interest

The authors declare that the research was conducted in the absence of any commercial or financial relationships that could be construed as a potential conflict of interest.

## Publisher’s note

All claims expressed in this article are solely those of the authors and do not necessarily represent those of their affiliated organizations, or those of the publisher, the editors and the reviewers. Any product that may be evaluated in this article, or claim that may be made by its manufacturer, is not guaranteed or endorsed by the publisher.

## References

[ref1] Abdelrahman M. (2022). Personality traits, risk perception, and protective behaviors of Arab residents of Qatar during the COVID-19 pandemic. Int. J. Ment. Heal. Addict. 20, 237–248. doi: 10.1007/s11469-020-00352-7, PMID: 32837433 PMC7307935

[ref2] Agag G. Abdelmoety Z. H. Eid R. (2023). Understanding the factors affecting travel avoidance behavior during the COVID-19 pandemic: findings from a mixed method approach. J. Travel Res. 2110. doi: 10.1177/00472875231182110 [Epub ahead of print].

[ref3] Airak S. Sukor N. S. A. Rahman N. A. (2023). Travel behaviour changes and risk perception during COVID-19: a case study of Malaysia. Transp. Res. Interdiscip. Perspect. 18:100784. doi: 10.1016/j.trip.2023.100784, PMID: 36844954 PMC9939401

[ref4] Akritidis J. McGuinness S. L. Leder K. (2022). University students’ travel risk perceptions and risk-taking willingness during the COVID-19 pandemic: a cross-sectional study. Travel Med. Infect. Dis. 51:102486. doi: 10.1016/j.tmaid.2022.102486, PMID: 36374786 PMC9617625

[ref5] Albarracín D. Wyer R. S. (2000). The cognitive impact of past behavior: influences on beliefs, attitudes, and future behavioral decisions. J. Pers. Soc. Psychol. 79, 5–22. doi: 10.1037//0022-3514.79.1.5, PMID: 10909874 PMC4807731

[ref6] Arnold M. Goldschmitt M. Rigotti T. (2023). Dealing with information overload: a comprehensive review [review]. Front. Psychol. 14:1122200. doi: 10.3389/fpsyg.2023.1122200, PMID: 37416535 PMC10322198

[ref7] Bae S. Y. Chang P.-J. (2020). The effect of coronavirus disease-19 (COVID-19) risk perception on behavioural intention towards ‘untact’ tourism in South Korea during the first wave of the pandemic (march 2020). Curr. Issue Tour. 24, 1017–1035. doi: 10.1080/13683500.2020.1798895

[ref8] Bezjian-Avery A. A. Calder B. Iacobucci D. (1998). New media interactive advertising vs. traditional advertising. J. Advert. Res. 38, 23–32.

[ref9] Bhati A. S. Mohammadi Z. Agarwal M. Kamble Z. Donough-Tan G. (2020). Motivating or manipulating: the influence of health-protective behaviour and media engagement on post-COVID-19 travel. Curr. Issue Tour. 24, 2088–2092. doi: 10.1080/13683500.2020.1819970

[ref10] Bish A. Michie S. (2010). Demographic and attitudinal determinants of protective behaviours during a pandemic: a review. Br. J. Health Psychol. 15, 797–824. doi: 10.1348/135910710X485826 20109274 PMC7185452

[ref11] Borowski E. Chen Y. Mahmassani H. (2020). Social media effects on sustainable mobility opinion diffusion: model framework and implications for behavior change. Travel Behav. Soc. 19, 170–183. doi: 10.1016/j.tbs.2020.01.003

[ref12] Cahyanto I. Liu-Lastres B. (2020). Risk perception, media exposure, and visitor’s behavior responses to Florida red tide. J. Travel Tour. Mark. 37, 447–459. doi: 10.1080/10548408.2020.1783426

[ref13] Cahyanto I. Wiblishauser M. Pennington-Gray L. Schroeder A. (2016). The dynamics of travel avoidance: the case of Ebola in the U.S. tourism management. Perspective 20, 195–203. doi: 10.1016/j.tmp.2016.09.004, PMID: 32289007 PMC7147605

[ref14] Chien P. M. Sharifpour M. Ritchie B. W. Watson B. (2016). Travelers’ health risk perceptions and protective behavior: a psychological approach. J. Travel Res. 56, 744–759. doi: 10.1177/0047287516665479

[ref15] Chua B.-L. Al-Ansi A. Lee M. J. Han H. (2021). Impact of health risk perception on avoidance of international travel in the wake of a pandemic. Curr. Issue Tour. 24, 985–1002. doi: 10.1080/13683500.2020.1829570

[ref16] Courbet D. Fourquet-Courbet M.-P. Basile-Commaille É. Bernard P. Pascual-Espuny C. Kouadio P.. (2022). Media as a source of coping and social, psychological and hedonic well-being: a longitudinal qualitative study during the COVID-19 pandemic. Media Psychol. 26, 306–335. doi: 10.1080/15213269.2022.2142244

[ref17] Dryhurst S. Schneider C. R. Kerr J. Freeman A. L. J. Recchia G. van der Bles A. M.. (2020). Risk perceptions of COVID-19 around the world. J. Risk Res. 23, 994–1006. doi: 10.1080/13669877.2020.1758193

[ref18] Du E. Chen E. Liu J. Zheng C. (2021). How do social media and individual behaviors affect epidemic transmission and control? Sci. Total Environ. 761:144114. doi: 10.1016/j.scitotenv.2020.144114 33360131 PMC7834887

[ref19] Flew T. (2008). New media: an introduction (third edition). Australia: Oxford University Press.

[ref20] Floyd D. Prentice-Du S. Rogers R. W. (2000). A Meta-analysis of research on protection motivation theory. J. Appl. Soc. Psychol. 30, 407–429. doi: 10.1111/j.1559-1816.2000.tb02323.x

[ref21] Frh H. (2017). “Risk perception as media effect” in The international encyclopedia of media effects. ed. Rössler P., Wiley 1–8.

[ref22] Gibbs H. Liu Y. Pearson C. A. B. Jarvis C. I. Grundy C. Quilty B. J.. (2020). Changing travel patterns in China during the early stages of the COVID-19 pandemic. Nat. Commun. 11:5012. doi: 10.1038/s41467-020-18783-0 33024096 PMC7538915

[ref23] Giustini D. Ali S. M. Fraser M. Boulos M. N. K. (2018). Effective uses of social media in public health and medicine: a systematic review of systematic reviews. Online J. Public Health Inform. 10:e215. doi: 10.5210/ojphi.v10i2.8270, PMID: 30349633 PMC6194097

[ref24] Golets A. Farias J. Pilati R. Costa H. (2021). COVID-19 pandemic and tourism: the impact of health risk perception and intolerance of uncertainty on travel intentions. Curr. Psychol. 42, 2500–2513. doi: 10.1007/s12144-021-02282-6, PMID: 34539156 PMC8436199

[ref25] Hansen M. F. Sørensen P. K. Sørensen A. E. Krogfelt K. A. (2023). Can protection motivation theory predict protective behavior against ticks? BMC Public Health 23:1214. doi: 10.1186/s12889-023-16125-5, PMID: 37349761 PMC10286392

[ref26] Hennig-Thurau T. Malthouse E. C. Friege C. Gensler S. Lobschat L. Rangaswamy A.. (2010). The impact of new media on customer relationships. J. Serv. Res. 13, 311–330. doi: 10.1177/1094670510375460

[ref27] Hotle S. Murray-Tuite P. Singh K. (2020). Influenza risk perception and travel-related health protection behavior in the US: insights for the aftermath of the COVID-19 outbreak. Transp. Res. Interdiscip. Perspect. 5:100127. doi: 10.1016/j.trip.2020.100127, PMID: 34171017 PMC7211591

[ref28] Huang Y. Lai Q. Ling F. (2018). Influence of social media on tourists' tourism intention: empirical research based on perception of destination image. Res. Dev. Market 34, 1327–1331.

[ref29] Iacobucci D. (2010). Structural equations modeling: fit indices, sample size, and advanced topics. J. Consum. Psychol. 20, 90–98. doi: 10.1016/j.jcps.2009.09.003

[ref30] Jiang X. Qin J. Gao J. Gossage M. G. (2022). How tourists' perception affects travel intention: mechanism pathways and boundary conditions. Front. Psychol. 13:821364. doi: 10.3389/fpsyg.2022.821364, PMID: 35783752 PMC9245517

[ref31] Johnson B. B. Mayorga M. Kim B. (2023). Americans’ COVID-19 risk perceptions and risk perception predictors changed over time. J. Risk Res. 26, 815–835. doi: 10.1080/13669877.2023.2208149

[ref32] Kim J. Yang K. Min J. White B. (2022). Hope, fear, and consumer behavioral change amid COVID-19: application of protection motivation theory. Int. J. Consum. Stud. 46, 558–574. doi: 10.1111/ijcs.12700, PMID: 34220343 PMC8237022

[ref33] Koo C. Joun Y. Han H. Chung N. (2016). A structural model for destination travel intention as a media exposure. Int. J. Contemp. Hosp. Manag. 28, 1338–1360. doi: 10.1108/IJCHM-07-2014-0354

[ref34] Kowalski R. M. Black K. J. (2021). Protection motivation and the COVID-19 virus. Health Commun. 36, 15–22. doi: 10.1080/10410236.2020.1847448 33190547

[ref35] Lachapelle U. Jean-Germain F. (2019). Personal use of the internet and travel: evidence from the Canadian general social Survey’s 2010 time use module. Travel Behav. Soc. 14, 81–91. doi: 10.1016/j.tbs.2018.10.002

[ref36] Lebrun A.-M. Corbel R. Bouchet P. (2021). Impacts of Covid-19 on travel intention for summer 2020: a trend in proximity tourism mediated by an attitude towards Covid-19. Serv. Bus. 16, 469–501. doi: 10.1007/s11628-021-00450-z

[ref38] Liebig J. Najeebullah K. Jurdak R. Shoghri A. E. Paini D. (2021). Should international borders re-open? The impact of travel restrictions on COVID-19 importation risk. BMC Public Health 21:1573. doi: 10.1186/s12889-021-11616-9, PMID: 34416860 PMC8378112

[ref39] Liu L. Xie J. Li K. Ji S. (2020). Exploring how media influence preventive behavior and excessive preventive intention during the COVID-19 pandemic in China. Int. J. Environ. Res. Public Health 17:7990. doi: 10.3390/ijerph17217990, PMID: 33143145 PMC7663107

[ref40] Liu M. Zhang H. Huang H. (2020). Media exposure to COVID-19 information, risk perception, social and geographical proximity, and self-rated anxiety in China. BMC Public Health 20:1649. doi: 10.1186/s12889-020-09761-8, PMID: 33148201 PMC7609828

[ref9001] Li W. Zhang C. Zhou X., and Jin Q. (2023). Dynamic multi-view group preference learning for group behavior prediction in social networks. Expert Syst. Appl. 120553. doi: 10.1016/j.eswa.2023.120553

[ref37] Li Z. Zhang M. Wei R. Zhu Y. (2021). WeChat use and altruistic behavior in the COVID-19 crisis:the mediated effects of risk perception and public trust. Int. Press 5, 6–22. doi: 10.13495/j.cnki.cjjc.2021.05.001

[ref41] Luo L. Wang X. (2021). How health communication via social media and television shapes users' preventive behavior of COVID-19: an empirical study based on the theory of risk perception. Med. Soc. 34, 106–112.

[ref42] Ma L. Cao J. (2019). How perceptions mediate the effects of the built environment on travel behavior? Transportation 46, 175–197. doi: 10.1007/s11116-017-9800-4

[ref43] Ma Z. R. Idris S. Pan Q. W. Baloch Z. (2021). COVID-19 knowledge, risk perception, and information sources among Chinese population. World J. Psychiatry 11, 181–200. doi: 10.5498/wjp.v11.i5.181, PMID: 34046314 PMC8134868

[ref44] Meng Y. Khan A. Bibi S. Wu H. Lee Y. Chen W. (2021). The effects of COVID-19 risk perception on travel intention: evidence from Chinese travelers. Front. Psychol. 12:655860. doi: 10.3389/fpsyg.2021.655860, PMID: 34335367 PMC8322978

[ref45] Nazione S. Perrault E. Pace K. (2021). Impact of information exposure on perceived risk, efficacy, and preventative behaviors at the beginning of the COVID-19 pandemic in the United States. Health Commun. 36, 23–31. doi: 10.1080/10410236.2020.1847446, PMID: 33183090

[ref46] Neuburger L. Egger R. (2020). Travel risk perception and travel behaviour during the COVID-19 pandemic 2020: a case study of the DACH region. Curr. Issue Tour. 24, 1003–1016. doi: 10.1080/13683500.2020.1803807

[ref47] Oh S. H. Lee S. Y. Han C. (2021). The effects of social media use on preventive behaviors during infectious disease outbreaks: the mediating role of self-relevant emotions and public risk perception. Health Commun. 36, 972–981. doi: 10.1080/10410236.2020.1724639, PMID: 32064932

[ref48] Olagoke A. A. Olagoke O. O. Hughes A. M. (2020). Exposure to coronavirus news on mainstream media: the role of risk perceptions and depression. Br. J. Health Psychol. 25, 865–874. doi: 10.1111/bjhp.12427 32415914 PMC7267047

[ref49] Pakpour A. H. Zeballos Rivas D. R. Lopez Jaldin M. L. Nina Canaviri B. Portugal Escalante L. F. Alanes Fernández A. M. C.. (2021). Social media exposure, risk perception, preventive behaviors and attitudes during the COVID-19 epidemic in La Paz, Bolivia: a cross sectional study. PLoS One 16, e0245859–e0245812. doi: 10.1371/journal.pone.0245859, PMID: 33481945 PMC7822287

[ref50] Papagiannidis S. Alamanos E. Bourlakis M. Dennis C. (2022). The pandemic consumer response: a stockpiling perspective and shopping channel preferences. Br. J. Manag. 34, 664–691. doi: 10.1111/1467-8551.12616

[ref51] Pidgeon N. (2010). “The social amplification of risk framework (SARF): theory, critiques, and policy implications” in Risk communication and public health. *2nd edn*. eds. Bennett P. Calman K. Curtis S. Fischbacher-Smith D. (Oxford: Oxford University Press).

[ref52] Popiołek M. Hapek M. Barańska M. (2021). Infodemia – an analysis of fake news in polish news portals and traditional media during the coronavirus pandemic. Commun. Soc. 34, 81–98. doi: 10.15581/003.34.4.81-98

[ref53] Raina D. I. Nasir T. Qazi T. (2022). Impact assessment of Covid-19 pandemic on perception and travel behavior of tourists with strategies for resilient recovery of Jammu and Kashmir, India. J. Tour. Insights 12:1253. doi: 10.9707/2328-0824.1253

[ref9002] Ribeiro M. A. Pinto P. Silva J. A., and Woosnam K. M. (2017). Residents’ attitudes and the adoption of pro-tourism behaviours: the case of developing island countries. Tour. Manag. 61, 523–537. doi: 10.1016/j.tourman.2017.03.004

[ref54] Rosen L. D. Whaling K. Carrier L. M. Cheever N. A. Rokkum J. (2013). The media and technology usage and attitudes scale: an empirical investigation. Comput. Hum. Behav. 29, 2501–2511. doi: 10.1016/j.chb.2013.06.006, PMID: 25722534 PMC4338964

[ref55] Salman A. Ibrahim F. Abdullah M. Y. Mustaffa N. Mahbob M. H. (2011). The impact of new media on traditional mainstream mass media. Innov. J. 16, 1–11.

[ref56] Sánchez-Cañizares S. M. Cabeza-Ramírez L. J. Muñoz-Fernández G. Fuentes-García F. J. (2021). Impact of the perceived risk from Covid-19 on intention to travel. Curr. Issue Tour. 24, 970–984. doi: 10.1080/13683500.2020.1829571

[ref57] Savadori L. Lauriola M. (2021). Risk perception and protective behaviors during the rise of the COVID-19 outbreak in Italy. Front. Psychol. 11:577331. doi: 10.3389/fpsyg.2020.577331, PMID: 33519593 PMC7838090

[ref58] Schneiders P. Kist E. L. Stark B. (2022). “Media Use” in Handbook of media and communication economics: a European perspective. eds. Krone J. Pellegrini T. (Wiesbaden: Springer), 1–28.

[ref59] Selem K. M. Sinha R. Khalid R. Raza M. Shahidul Islam M. (2023). Trade-off between future travel avoidance and self-protectiveness post-COVID-19: the roles of adventurousness and safety-seeking tendency. J. Hosp. Tour. Insights. doi: 10.1108/JHTI-09-2022-0432 [Epub ahead of print].

[ref60] Seo M. (2019). Amplifying panic and facilitating prevention: multifaceted effects of traditional and social media use during the 2015 MERS crisis in South Korea. Journal. Mass Commun. Q. 98, 221–240. doi: 10.1177/1077699019857693

[ref61] Seyfi S. Hall C. M. Shabani B. (2020). COVID-19 and international travel restrictions: the geopolitics of health and tourism. Tour. Geogr. 2, 1–17.

[ref62] Siapera E. (2018). Understanding new media. 2nd Edn. UK: SAGE Publications Ltd.

[ref63] Siegrist M. Luchsinger L. Bearth A. (2021). The impact of trust and risk perception on the acceptance of measures to reduce COVID-19 cases. Risk Anal. 41, 787–800. doi: 10.1111/risa.13675, PMID: 33438218 PMC8014821

[ref64] SMACT. (2021). Urgent notice on further strengthening the prevention and control of epidemic situation in the cultural and tourism industries of Shanghai. Available at: http://whlyj.sh.gov.cn/wlyw/20210806/249a316248ce4047a27af41e4ace9081.html

[ref65] Stever G. S. Giles D. C. Cohen D. Myers M. E. (2021). Understanding media psychology. New York: Routledge.

[ref66] Tomczyk S. Rahn M. Schmidt S. (2022). Sociodemographic and psychosocial profiles of multi-media use for risk communication in the general population. Int. J. Environ. Res. Public Health 19:12777. doi: 10.3390/ijerph191912777, PMID: 36232075 PMC9564902

[ref67] Vai B. Cazzetta S. Ghiglino D. Parenti L. Saibene G. Toti M.. (2020). Risk perception and media in shaping protective behaviors: insights from the early phase of COVID-19 Italian outbreak. Front. Psychol. 11:563426. doi: 10.3389/fpsyg.2020.563426, PMID: 33250809 PMC7674945

[ref68] Valkenburg P. M. Peter J. Walther J. B. (2016). Media effects: theory and research. Annu. Rev. Psychol. 67, 315–338. doi: 10.1146/annurev-psych-122414-033608 26331344

[ref69] Villacé-Molinero T. Fernández-Muñoz J. J. Orea-Giner A. Fuentes-Moraleda L. (2021). Understanding the new post-COVID-19 risk scenario: outlooks and challenges for a new era of tourism. Tour. Manag. 86:104324. doi: 10.1016/j.tourman.2021.104324, PMID: 36540617 PMC9756354

[ref70] Wang J. Liu-Lastres B. Ritchie B. W. Mills D. J. (2019). Travellers' self-protections against health risks: an application of the full protection motivation theory. Ann. Tour. Res. 78:102743. doi: 10.1016/j.annals.2019.102743

[ref71] Wang J. Zheng P. (2019). A study on the media use behavior, trust, and behavioral intentions of domestic potential visitors. Tour. Sci. 33, 59–72. doi: 10.16323/j.cnki.lykx.2019.02.005

[ref72] Weerd W. Timmermans D. Beaujean D. Oudhoff J. Steenbergen J. E. (2011). Monitoring the level of government trust, risk perception and intention of the general public to adopt protective measures during the influenza a (H1N1) pandemic in the Netherlands. BMC Public Health 11:575. doi: 10.1186/1471-2458-11-575, PMID: 21771296 PMC3152536

[ref73] Wen J. Kozak M. Yang S. Liu F. (2021). COVID-19: potential effects on Chinese citizens’ lifestyle and travel. Tour. Rev. 76, 74–87. doi: 10.1108/TR-03-2020-0110

[ref74] Woo J. Choi J. Y. Shin J. Lee J. (2014). The effect of new media on consumer media usage: an empirical study in South Korea. Technol. Forecast. Soc. Chang. 89, 3–11. doi: 10.1016/j.techfore.2014.09.001

[ref77] Wurtele S. Maddux J. (1987). Relative contributions of protection motivation theory components in predicting exercise intentions and behavior. Health Psychol. 6, 453–466. doi: 10.1037/0278-6133.6.5.453 3678171

[ref75] Wu X. Li X. (2017). Effects of mass media exposure and social network site involvement on risk perception of and precautionary behavior toward the haze issue in China. Int. J. Commun. 11, 3975–3997.

[ref76] Wu Y. Yang S. Liu D. (2023). The effect of social media influencer marketing on sustainable food purchase: perspectives from multi-group SEM and ANN analysis. J. Clean. Prod. 416:137890. doi: 10.1016/j.jclepro.2023.137890

[ref79] Xie J. Liu L. (2022). Identifying features of source and message that influence the retweeting of health information on social media during the COVID-19 pandemic. BMC Public Health 22:805. doi: 10.1186/s12889-022-13213-w, PMID: 35459154 PMC9026044

[ref78] Xie Q. (2021). The influence of media use and panic on social media Users' curatorial news of epidemic. Int. Press 5, 43–64. doi: 10.13495/j.cnki.cjjc.2021.05.003

[ref80] Xue L. Jing S. Sun W. Liu M. Peng Z. Zhu H. (2021). Evaluating the impact of the travel ban within mainland China on the epidemic of the COVID-19. Int. J. Infect. Dis. 107, 278–283. doi: 10.1016/j.ijid.2021.03.088, PMID: 33838344 PMC8024219

[ref81] Yang S. Isa S. M. Ramayah T. (2022a). Does uncertainty avoidance moderate the effect of self-congruity on revisit intention? A two-city (Auckland and Glasgow) investigation. J. Destin. Mark. Manag. 24:100703. doi: 10.1016/j.jdmm.2022.100703

[ref82] Yang S. Isa S. M. Ramayah T. (2022b). How are destination image and travel intention influenced by misleading media coverage? Consequences of COVID-19 outbreak in China. Vision 26, 80–89. doi: 10.1177/0972262921993245

[ref83] Yang S. Isa S. M. Ramayah T. Wen J. Goh E. (2022c). Developing an extended model of self-congruity to predict Chinese tourists' revisit intentions to New Zealand: the moderating role of gender. Asia Pac. J. Mark. Logist. 34, 1459–1481. doi: 10.1108/APJML-05-2021-0346

[ref84] Yoo W. Choi D. H. Park K. (2016). The effects of SNS communication: how expressing and receiving information predict MERS-preventive behavioral intentions in South Korea. Comput. Hum. Behav. 62, 34–43. doi: 10.1016/j.chb.2016.03.058, PMID: 32288174 PMC7127459

[ref85] Zhou S. (2022). Impact of pandemic proximity and media use on risk perception during COVID-19 in China. Geomat. Nat. Haz. Risk 13, 591–609. doi: 10.1080/19475705.2021.2003875

