# Supplementary material for: The impact of the intensity of media use on potential tourists’ risk perception and travel protective behavioral intentions in COVID-19
Source: Front Psychol. 2023 Aug 29;14:1201481. doi: 10.3389/fpsyg.2023.1201481 (PMC10495595; doi:10.3389/fpsyg.2023.1201481)
Supplement: Supplementary file 1 [file Data_Sheet_1.docx]

Appendix 1

Table 1.1 Gender multi-group analysis

| **Regression Weights:** | **Male - Unconstrained** | | | | **Female - Unconstrained** | | | |
| --- | --- | --- | --- | --- | --- | --- | --- | --- |
|  | Estimate | S.E. | C.R. | P | Estimate | S.E. | C.R. | P |
| ERP<---NMU | 0.646 | 0.13 | 4.989 | *** | 0.354 | 0.076 | 4.667 | *** |
| ERP<---TMU | -0.437 | 0.085 | -5.127 | *** | -0.118 | 0.056 | -2.11 | ** |
| TI <---TMU | 0.064 | 0.061 | 1.052 | 0.293 | -0.053 | 0.042 | -1.268 | 0.205 |
| TI <---NMU | 0.263 | 0.092 | 2.861 | *** | 0.25 | 0.061 | 4.065 | *** |
| TI <---ERP | 0.346 | 0.072 | 4.822 | *** | 0.299 | 0.064 | 4.689 | *** |

Table 1.2 Age multi-group analysis

| **Regression Weights:** | **<18 - Unconstrained** | | | | **>=18, < 25 - Unconstrained** | | | |
| --- | --- | --- | --- | --- | --- | --- | --- | --- |
|  | Estimate | S.E. | C.R. | P | Estimate | S.E. | C.R. | P |
| ERP<---NMU | - | - | - | - | 0.506 | 0.094 | 5.371 | *** |
| ERP<---TMU | - | - | - | - | -0.212 | 0.067 | -3.142 | *** |
| TI <---TMU | - | - | - | - | -0.028 | 0.053 | -0.536 | 0.592 |
| TI <---NMU | - | - | - | - | 0.219 | 0.078 | 2.798 | *** |
| TI <---ERP | - | - | - | - | 0.409 | 0.077 | 5.285 | *** |
| **Regression Weights:** | **>= 25, < 50 - Unconstrained** | | | | **>= 50 - Unconstrained** | | | |
|  | Estimate | S.E. | C.R. | P | Estimate | S.E. | C.R. | P |
| ERP<---NMU | 0.453 | 0.128 | 3.529 | *** | 0.167 | 0.286 | 0.584 | 0.559 |
| ERP<---TMU | -0.269 | 0.079 | -3.393 | *** | -0.665 | 0.328 | -2.029 | ** |
| TI <---TMU | -0.059 | 0.047 | -1.259 | 0.208 | 0.233 | 0.189 | 1.232 | 0.218 |
| TI <---NMU | 0.336 | 0.086 | 3.925 | *** | 0.376 | 0.186 | 2.024 | ** |
| TI <---ERP | 0.269 | 0.063 | 4.286 | *** | 0.197 | 0.147 | 1.335 | 0.182 |

Table 1.3 Occupation multi-group analysis

| **Regression Weights:** | **Student - Unconstrained** | | | | **Government and public institutions - Unconstrained** | | | |
| --- | --- | --- | --- | --- | --- | --- | --- | --- |
|  | Estimate | S.E. | C.R. | P | Estimate | S.E. | C.R. | P |
| ERP<---NMU | 0.406 | 0.084 | 4.832 | *** | 0.583 | 0.38 | 1.535 | 0.125 |
| ERP<---TMU | -0.132 | 0.074 | -1.784 | * | -0.618 | 0.18 | -3.424 | *** |
| TI <---TMU | -0.005 | 0.056 | -0.085 | 0.932 | -0.040 | 0.208 | -0.191 | 0.849 |
| TI <---NMU | 0.101 | 0.067 | 1.497 | 0.134 | 0.779 | 0.352 | 2.213 | ** |
| TI <---ERP | 0.437 | 0.083 | 5.246 | *** | 0.323 | 0.211 | 1.532 | 0.126 |
| **Regression Weights:** | **Enterprise - Unconstrained** | | | | **Others - Unconstrained** | | | |
|  | Estimate | S.E. | C.R. | P | Estimate | S.E. | C.R. | P |
| ERP<---NMU | 0.373 | 0.129 | 2.884 | *** | 0.586 | 0.176 | 3.323 | *** |
| ERP<---TMU | -0.236 | 0.077 | -3.047 | *** | -0.268 | 0.149 | -1.797 | * |
| TI <---TMU | -0.035 | 0.050 | -0.700 | 0.484 | 0.050 | 0.093 | 0.532 | 0.594 |
| TI <---NMU | 0.355 | 0.100 | 3.551 | *** | 0.354 | 0.122 | 2.893 | *** |
| TI <---ERP | 0.290 | 0.079 | 3.671 | *** | 0.144 | 0.083 | 1.742 | * |

Table 1.4 Education multi-group analysis

| **Regression Weights:** | **Junior high school and below - Unconstrained** | | | | **High school or technical secondary school**  **- Unconstrained** | | | |
| --- | --- | --- | --- | --- | --- | --- | --- | --- |
|  | Estimate | S.E. | C.R. | P | Estimate | S.E. | C.R. | P |
| ERP<---NMU | 0.264 | 0.137 | 1.927 | ** | 0.219 | 0.132 | 1.655 | * |
| ERP<---TMU | -0.332 | 0.198 | -1.671 | * | -0.257 | 0.156 | -1.650 | * |
| TI <---TMU | -0.075 | 0.201 | -0.374 | 0.708 | 0.302 | 0.110 | 2.747 | *** |
| TI <---NMU | 0.435 | 0.158 | 2.762 | *** | -0.021 | 0.081 | -0.255 | 0.799 |
| TI <---ERP | 0.327 | 0.215 | 1.516 | 0.129 | 0.505 | 0.143 | 3.530 | *** |
| **Regression Weights:** | **College and undergraduate - Unconstrained** | | | | **Master or above - Unconstrained** | | | |
|  | Estimate | S.E. | C.R. | P | Estimate | S.E. | C.R. | P |
| ERP<---NMU | 0.896 | 0.161 | 5.569 | *** | 0.196 | 0.097 | 2.016 | *** |
| ERP<---TMU | -0.276 | 0.072 | -3.846 | *** | -0.285 | 0.088 | -3.227 | *** |
| TI <---TMU | -0.055 | 0.050 | -1.100 | 0.271 | -0.066 | 0.058 | -1.126 | 0.260 |
| TI <---NMU | 0.489 | 0.121 | 4.037 | *** | 0.132 | 0.064 | 2.061 | *** |
| TI <---ERP | 0.258 | 0.061 | 4.203 | *** | 0.240 | 0.081 | 2.960 | *** |

Table 1.5 Income multi-group analysis (Rmb Yuan/month)

| **Regression Weights:** | **< 2480 - Unconstrained** | | | | **2480-5000 - Unconstrained** | | | |
| --- | --- | --- | --- | --- | --- | --- | --- | --- |
|  | Estimate | S.E. | C.R. | P | Estimate | S.E. | C.R. | P |
| ERP<---NMU | 0.420 | 0.077 | 5.446 | *** | 0.462 | 0.166 | 2.784 | *** |
| ERP<---TMU | -0.209 | 0.069 | -3.051 | *** | -0.221 | 0.114 | -1.936 | * |
| TI <---TMU | 0.004 | 0.055 | 0.081 | 0.935 | -0.042 | 0.091 | -0.457 | 0.647 |
| TI <---NMU | 0.137 | 0.065 | 2.105 | ** | 0.278 | 0.139 | 2.000 | ** |
| TI <---ERP | 0.554 | 0.085 | 6.501 | *** | 0.274 | 0.115 | 2.377 | ** |
| **Regression Weights:** | **5000-10000 - Unconstrained** | | | | **>10000 - Unconstrained** | | | |
|  | Estimate | S.E. | C.R. | P | Estimate | S.E. | C.R. | P |
| ERP<---NMU | 0.552 | 0.196 | 2.817 | *** | 0.065 | 0.187 | 0.348 | 0.728 |
| ERP<---TMU | -0.207 | 0.102 | -2.027 | ** | -0.372 | 0.129 | -2.893 | *** |
| TI <---TMU | -0.011 | 0.049 | -0.233 | 0.816 | -0.003 | 0.047 | -0.062 | 0.950 |
| TI <---NMU | 0.393 | 0.117 | 3.367 | *** | 0.043 | 0.066 | 0.650 | * |
| TI <---ERP | 0.110 | 0.052 | 2.104 | ** | 0.342 | 0.188 | 1.819 | * |

Table 1.6 With or without travel experiences multi-group analysis

| **Regression Weights:** | **Traveled - Unconstrained** | | | | **No travel - Unconstrained** | | | |
| --- | --- | --- | --- | --- | --- | --- | --- | --- |
|  | Estimate | S.E. | C.R. | P | Estimate | S.E. | C.R. | P |
| ERP<---NMU | 0.354 | 0.120 | 2.953 | *** | 0.501 | 0.084 | 5.956 | *** |
| ERP<---TMU | -0.160 | 0.094 | -1.702 | *** | -0.275 | 0.054 | -5.058 | *** |
| TI <---TMU | -0.05 | 0.102 | -0.497 | 0.619 | 0.028 | 0.034 | 0.826 | 0.409 |
| TI <---NMU | 0.278 | 0.139 | 1.992 | *** | 0.195 | 0.054 | 3.589 | *** |
| TI <---ERP | 0.566 | 0.239 | 2.370 | *** | 0.306 | 0.044 | 7.026 | *** |
